# Frataxin mRNA Isoforms in FRDA Patients and Normal Subjects: Effect of Tocotrienol Supplementation

**DOI:** 10.1155/2013/276808

**Published:** 2013-09-23

**Authors:** Provvidenza Maria Abruzzo, Marina Marini, Alessandra Bolotta, Gemma Malisardi, Stefano Manfredini, Alessandro Ghezzo, Antonella Pini, Gianluca Tasco, Rita Casadio

**Affiliations:** ^1^Department of Experimental, Diagnostic and Specialty Medicine, University of Bologna, 40126 Bologna, Italy; ^2^Department of Pharmaceutical Sciences, University of Ferrara, 44100 Ferrara, Italy; ^3^ANFFAS ONLUS Macerata, 62100 Macerata, Italy; ^4^Child Neurology and Psychiatry Unit, IRCCS Institute of Neurological Sciences of Bologna, Bologna, 40100 Bologna, Italy; ^5^Biocomputing Group, CIRI-Health Science and Technology, Department of Biology, Bologna 40126, Italy

## Abstract

Friedreich's ataxia (FRDA) is caused by deficient expression of the mitochondrial protein frataxin involved in the formation of iron-sulphur complexes and by consequent oxidative stress. We analysed low-dose tocotrienol supplementation effects on the expression of the three splice variant isoforms (*FXN-1*, *FXN-2*, and *FXN-3*) in mononuclear blood cells of FRDA patients and healthy subjects. In FRDA patients, tocotrienol leads to a specific and significant increase of *FXN-3* expression while not affecting *FXN-1* and *FXN-2* expression. Since no structural and functional details were available for FNX-2 and FXN-3, 3D models were built. FXN-1, the canonical isoform, was then docked on the human iron-sulphur complex, and functional interactions were computed; when FXN-1 was replaced by FXN-2 or FNX-3, we found that the interactions were maintained, thus suggesting a possible biological role for both isoforms in human cells. Finally, in order to evaluate whether tocotrienol enhancement of *FXN-3* was mediated by an increase in peroxisome proliferator-activated receptor-**γ** (PPARG), *PPARG* expression was evaluated. At a low dose of tocotrienol, the increase of *FXN-3* expression appeared to be independent of *PPARG* expression. Our data show that it is possible to modulate the mRNA expression of the minor frataxin isoforms and that they may have a functional role.

## 1. Introduction 

Friedreich's ataxia (FRDA) is the most common ataxia among Caucasians, with an estimated incidence of 1 : 40,000 individuals [1]. It is inherited as autosomal recessive disorder with onset typically in the second decade of life. The majority of FRDA patients suffer hypertrophic cardiomyopathy, which is the principal cause of death [2].

The gene responsible for FRDA, *FXN*, located on chromosome 9, is composed by seven exons (exons 1–4, 5a, 5b, and 6) and encodes for a mitochondrial protein, frataxin. Frataxin is expressed at much lower levels (5–30%) in FRDA patients compared with normal individuals [3]. In 98% of cases, frataxin downregulation is due to a homozygous GAA trinucleotide expansion in the first intron of the *FXN*, which is responsible for the transcriptional silencing of *FXN *gene [4, 5].

At present, three different transcripts of *FXN* gene are known. The major transcript (1.3 kb) is composed by five exons (exons 1–4 and 5a) and encodes for a 210-amino acid protein, named frataxin isoform A or FXN-1 (NM_000144; NP_000135). A minor alternative transcript, frataxin isoform B or FXN-3, containing the exon 5b in place of exon 5a (NM_001161706) was described [4]. Due to an in-frame stop codon in exon 5b, *FXN-3* mRNA encodes for a shorter protein of 171- amino acid (NP_001155178). FXN-1 and FXN-3 proteins share a sequence identity of 93%, showing a difference in sequence and length in the C-terminus portion. FXN-3 C-terminus contains 11 residues instead of 48 residues of the FXN-1 (RLTWLLWLFHP in place of SGPKRYDWTGKNWVYSHDGVSLHELLAAELTKALKTKLDLSSLAYSGK). In 2002, Pianese and coworkers identified a third splice variant of frataxin, namely, frataxin isoform A1 or FXN-2. Similarly to *FXN-1*, *FXN-2* transcript is composed by the exons 1–4 and 5a (NM_181425) [6]. *FXN-2* mRNA differs from *FXN-1* mRNA for an insertion of 8 bp due to an alternative splicing site at 5′ end of intron 4. The 8 bp insertion generates a frameshift that introduces a stop codon site, so that this transcript encodes for a shorter protein of 196 amino acids (NP_852090). After their first description, *FXN-2* and *FXN-3* transcripts were not studied in detail and no functional data about the protein isoforms have been reported. Recently, Xia and coworkers have identified two novel isoforms of frataxin specifically expressed in affected cerebellum and heart [7]. 

Frataxin is a well-conserved and ubiquitous protein; its expression levels are rather low, with the exception of tissues such as muscle, heart, and cerebellum. The three-dimensional structure of human [8], yeast [9], and bacterial [10] frataxin was obtained. They are strictly alike, with different elements of secondary structure conserved. Frataxin is characterized by a flat *β*-sheet platform supported by two parallel *α*-helices at N and C terminus. Frataxin protein contains two structural elements that are conserved in all frataxin orthologs: (i) the presence of acidic residues in the first *α*-helix (*α*-1) and *β*-strand (*β*-1) which form a negatively charged surface; (ii) a neutral flat area on the *β*-sheet surface that probably allows the interaction of protein partner(s) on frataxin [8].

It is known that frataxin is involved in different biological processes such as iron binding and storage, biogenesis of heme and of iron-sulphur (ISC/NSF1) cluster proteins, and iron chaperone activity [11–14]. It has been shown that frataxin is involved also in Fe detoxification, and therefore it may play a key role in the regulation of oxidative stress [15]. Other physiological functions of frataxin are still to be determined.

There are no effective treatments to prevent the neurodegenerative progression of FRDA; currently, FRDA patients regularly take idebenone, an analogue of coenzyme Q10, which exerts its beneficial effects by ameliorating cardiac and cerebellum function and modulating oxidative stress [16, 17]. However, recent research has shown that in a 6-month randomized, double-blind, controlled study, idebenone did not improve cardiac [18] or neurological [19] function. For that reason, researchers focused on new molecules with higher antioxidant, neuroprotective, and cardioprotective proprieties [20] and/or on agents able to increase cellular frataxin expression [21, 22].

Tocotrienol could be a promising compound in FRDA treatment. Tocotrienols belong to the family of vitamin E and, unlike tocopherol, contain three double bonds in their isoprenoid side chain and have a greater bioavailability. They exhibit strong antioxidant, anticancer, cardioprotective, and neuroprotective properties [23]. In a recent study, we have shown that, in five FRDA patients treated with idebenone and in five healthy volunteers, a two-month low-dose tocotrienol supplementation led to the decrease of oxidative stress indexes (M. Marini, submitted paper).

Anderson and colleagues have shown that tocotrienol is able to regulate directly the expression of IKBKAP whose alteration is responsible for a neurodegenerative disease, familial dysautonomia [24, 25].

The aim of this study was to evaluate, in FRDA patients regularly assuming idebenone and in healthy volunteers, the effect of two months tocotrienol supplementation on frataxin isoforms gene expression. Since frataxin is a ubiquitous protein, we evaluated its expression in mononuclear blood cells, as previously reported [5]. Moreover, in order to understand whether the less expressed FXN-2 and FXN-3 isoforms may have a possible role in the cell, a 3D model of both isoforms and a docking study between FXN-2 or FXN-3 and the human complex NFS1/ISCU were determined. 

## 2. Material and Methods

### 2.1. Patients and Healthy Subjects

Five FRDA patients and five healthy volunteers were enrolled after signing an informed consent form. The Child Neurology and Psychiatry Unit, IRCCS Institute of Neurological Sciences of Bologna, Bologna, Italy, enrolled all subjects in this study. This study was approved by the Ethical Committee of Regional Health Service (number 1635-08092011).

FRDA was diagnosed according to Harding 123 diagnostic criteria, and the genetic test showed an altered number of GAA repeats; in particular, the less expanded allele contains the following number of GAA repeats: patient 1, 830; patient 2, 625; patient 3, 560; patient 4, 360; patient 5, 621. Patients regularly assumed idebenone (5 mg/kg body weight/day), which was not discontinued during the study. 

Healthy volunteers, used as control, were age and sex matched with the FRDA patients; they were not affected by any neurological or psychiatric disease and were not under pharmacological treatment of any type nor were assuming food integrators.

### 2.2. Experimental Design

FRDA patients and healthy controls took a tocotrienol supplementation *per os* for two-month. Venous blood was collected before (presubjects) and at the end (postsubjects) of tocotrienol treatment. 

Tocotrienol dose used in this study (7 mg/kg body weight/day) was much lower than that estimated as no observed adverse effect level (NOEAL) in rats or humans [26, 27]. Neither FRDA patients, or healthy controls noticed adverse effects due to tocotrienol consumption. The tocotrienol mixture was designed by Ambrosialab s.r.l., a spin-off company of the University of Ferrara, Italy. It is a Palm Oil (Elaeis Guineensis) phytocomplex, prepared for the purposes of the study, as soft gel capsule formulation and labeled as OXI-3 (internal reference name ALAB103). It is composed by tocotrienols and tocopherols in the enantiomerically pure natural form (total tocotrienol and tocopherol: 256 mg/g, of which D-tocotrienol is 197 mg/g and D-tocopherol is 59 mg/g). 

### 2.3. Blood Mononuclear Cell Isolation

Venous blood (15 mL) from FRDA patients and controls was collected in Na-EDTA vacutainers and centrifuged 10 min at 800 ×g, in order to separate plasma, which was used for others studies. The blood was then diluted 1 : 1 with phosphate buffered saline (PBS), layered over HISTOPAQUE-1077 (Sigma, St. Louis, MO) and centrifuged 35 min at 453 g. Mononuclear cells were removed from the gradient interface and washed three times with PBS. The mononuclear cell pellet was dissolved with 1 mL of Trizol reagent (Invitrogen, Milan, Italy) and stored at −80°C before RNA extraction.

### 2.4. RNA Extraction and cDNA Synthesis

Total RNA was extracted from the Trizol suspension following the manufacturer's instructions and the paper by Chomczynski and Sacchi [28]. RNA quality and cDNA synthesis was assessed as previously described [29]. Briefly, RNA quality was measured by evaluation of 28S and 18S rRNA band sharpness after denaturing electrophoresis. Genomic DNA contamination was removed by digestion with RNase-free Deoxyribonuclease I (DNase I) (Amplification Grade DNase I, Sigma-Aldrich, St. Louis, MO), and its absence was assessed by PCR analysis using specific primers for HSP70 promoter (left primer: cgccatggagaccaacaccc; right primer: gcggttccctgctctctgtc). RNA purity and concentration were measured by spectrophotometer (Ultrospec 3000, Pharmacia Biotech, Cambridge, UK).

Equal amounts of total RNA were reverse transcribed using the iScript cDNA Synthesis Kit (Bio-Rad Laboratories, Hercules, CA) following the manufacturer's instructions. The cDNA thus obtained was stored at −20°C and used for semiquantitative and qRT-PCRs. 

The amount of protein recovered following Trizol extraction was insufficient for analysing frataxin protein expression.

### 2.5. Primer Design

Specific primer sequences for frataxin isoforms, for PPARG, and for two housekeeping genes, beta-actin and GAPDH, were designed with the help of two freely available software tools Primers3 and Amplify. 

**Figure 1:**
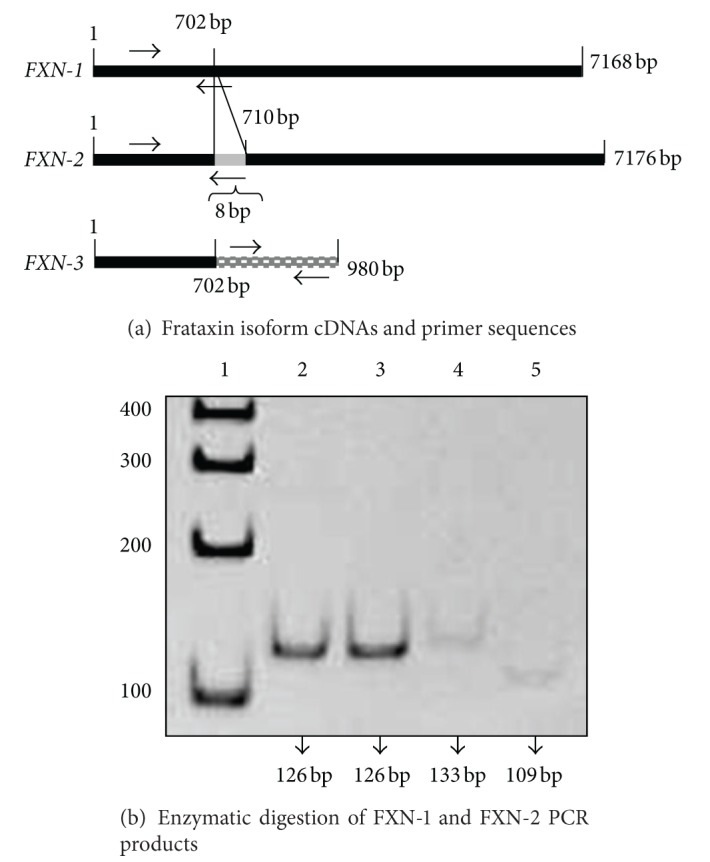
(a) A representative scheme of frataxin isoform cDNAs and primer sequences. Frataxin isoform 1 (*FXN-1*) and isoform 2 (*FXN-2*) share the left primer sequence, while the right primer was specific for each isoform. Frataxin isoform 3 (*FXN-3*) right primer sequence was designed in the region downstream the 702 base pair. (b) Enzymatic digestion of *FXN-1* and *FXN-2* PCR products. A representative polyacrylamide gel electrophoresis (10%) of *FXN-1* and *FXN-2* PCR products digested or undigested with BstNI restriction enzyme. Lane 1: DNA ladder; lane 2: undigested *FXN-1* PCR product; Lane 3: BstNI digested *FXN-1* PCR product; lane 4: undigested *FXN-2 *PCR product; lane 5: BstNI digested-*FXN-2* PCR product.

Figure 1(a) shows the cDNA frataxin isoforms and their respective primer sequences. In order to discriminate between *FXN-1* and *FXN-2* isoforms, the primer pairs shared the left primer, while the right primer was designed in the precise site where the two isoforms differ (i.e., in the region where the *FXN-2* isoform has 8 bp insertion). *FXN-3* primers were designed in the cDNA region following nucleotide 702. Primer sequences, summarized in Table 1 of supplementary data (see Supplementary Material available online at http://dx.doi.org/10.1155/2013/276808), were purchased from Sigma-GENOSYS (Sigma-Aldrich). To verify primer specificity, PCR products were subjected to both agarose gel electrophoresis and a melting curve analysis. Moreover, *FXN-1* and *FXN-2* primer specificity were evaluated by enzymatic digestion of PCR products obtained with semi-quantitative PCR.

### 2.6. Semiquantitative Polymerase Chain Reactions (PCR)

PCR analysis was performed using a Taq PCR core kit (Qiagen S.r.l., Milan, Italy), according to manufacturer's instructions. PCR conditions were as follows: a denaturing stage at 95°C for 5 min, 40 cycles at 95°C for 50 s, 62.5°C for 50 s, 72°C for 10 min, and a final stage extension at 72°C for 10 min. Positive and negative controls were used to check for contamination and unspecific amplifications. PCR products were analyzed on 2.5% agarose gel stained with ethidium bromide at 0.5 mg mL^−1^ after one hour of running at 70 mV.

### 2.7. Enzymatic Digestion of *FXN-1 *and *FXN-2* PCR Products

*FXN-1* and *FXN-2* PCR products, 126 bp and 133 bp, respectively, were digested with BstNI endonuclease (New England Biolabs, Milan, Italy), taking into account that its restriction site (5′ CCWGG 3′ and 3′ GGWCC 5′) is found exclusively in the *FXN-2* PCR product. To this purpose, *FXN-1* and *FXN-2* PCR products were incubated with 1X NEB Buffer 2, 1 mM BSA, and 1X BstNI in a final reaction volume of 20 *μ*L. The digestion was carried out at 60°C for 90 min. The digested fragments were separated by electrophoresis on 10% polyacrylamide gel along with undigested samples and were visualized by ethidium bromide staining.

### 2.8. Quantitative RT-PCR Analysis and Statistical Analysis

Quantitative RT-PCR was performed in a Bio-Rad CFX96 real-time thermal cycler using the SsoFast EvaGreen Supermix (Bio-Rad Laboratories, Hercules, CA). Using CFX Manager Software (Bio-Rad Laboratories, Hercules, CA) and qbase^plus^ (http://www.biogazelle.com/), data were analyzed with the 2^−ΔΔCT^ method [30], taking into account the efficiency of the real-time PCR reaction between 95% and 105% [31]. Quantitative RT-PCR evaluations were repeated at least twice. Data were evaluated by ANOVA test.

### 2.9. Modelling of Human FXN-2 and FXN-3 and of the Human NFS1/ISCU Complex

In order to build the 3D model of the human protein isoforms FXN-2 and FXN-3 (the targets), FXN-1 was adopted as a template. The human frataxin mature form (FXN-1) is determined at 1.80 Å (Swiss-Prot: FRDA_HUMAN; PDB code 1EKG [8]). Pairwise alignment of the different FXN-2 and FXN-3 with FXN-1 was generated with ClustalW2 (http://www.ebi.ac.uk/Tools/msa/clustalw2/) in an expert-driven mode, considering also the alignment of the secondary structure motifs to those of the template. Secondary structure prediction was performed with PSIPRED (http://bioinf.cs.ucl.ac.uk/psipred/). The different models were computed with Modeller version 9.8 [32]. For a given alignment, 20 model structures were built and evaluated with the programs PROCHECK [33] and ProSa [34] for stereo chemical and energetic validation, respectively, retaining only the model that better met the restrictions of both programs. Structural overlapping between FXN-1, FXN-2, and FXN-3, respectively, was done with MultiProt 3D (http://bioinfo3d.cs.tau.ac.il/MultiProt/). The 3D structure of the human complex NFS1/ISCU was computed on the basis of the available template of *E. coli* ISCS/ISCU (PDB code: 3LVL) [35]. The complex was computed with the same procedure described above. To calculate the tetrameric form, consisting of 2 chains of NFS1 and 2 chains of ISCU, the PDBePISA server (http://www.ebi.ac.uk/msd-srv/prot_int/) was adopted to predict the protein interfaces and consequently the likely quaternary structure of the protein complex.

### 2.10. Docking of the Different Human Frataxin Isoforms on the NSF1/ISCU Complex

Rigid molecular docking of the different human frataxin isoforms on the NSF1/ISCU complex was performed with Autodock version 4.2 Release 4.2.3 [36]. Five different atom-specific affinity maps were generated with autogrid4, (A, NA, C, OA and N) in addition to electrostatic and desolvation maps. The grid was centered on each ligand, consisting of 126 points (XYZ) and spacing of 0.475 Å that allows an unconstrained roto-translation of the ligand. For each isoform, twenty-five independent jobs were submitted by the Lamarckian genetic algorithm, with an initial population of 150 conformations, a cutoff of 27,000 generations, and with rates of mutation and crossover set to 0.02 and 0.8, respectively. The final solution was characterized by the lowest binding energy.

## 3. Results

### 3.1. *FXN-1* and *FXN-2* Primer Sequence Specificity

Having confirmed the primer specificity by both agarose gel electrophoresis and melting curve analysis (not shown), *FXN-1* and *FXN-2* PCR products, which differed in 8 bp (Figure 1(a)), were digested with the BstNI restriction enzyme. BstNI cleaves *FXN-2* PCR product (133 bp) in two fragments of 109 bp and 24 bp and leaves the *FXN-1* PCR product uncut. Undigested and digested fragments were separated on polyacrylamide gel (Figure 1(b)). As expected, *FXN-1* PCR product restricted with BstNI was undigested, while the 109 bp fragment shows the digestion operated by BstNI.

### 3.2. Semiquantitative PCR

**Figure 2:**
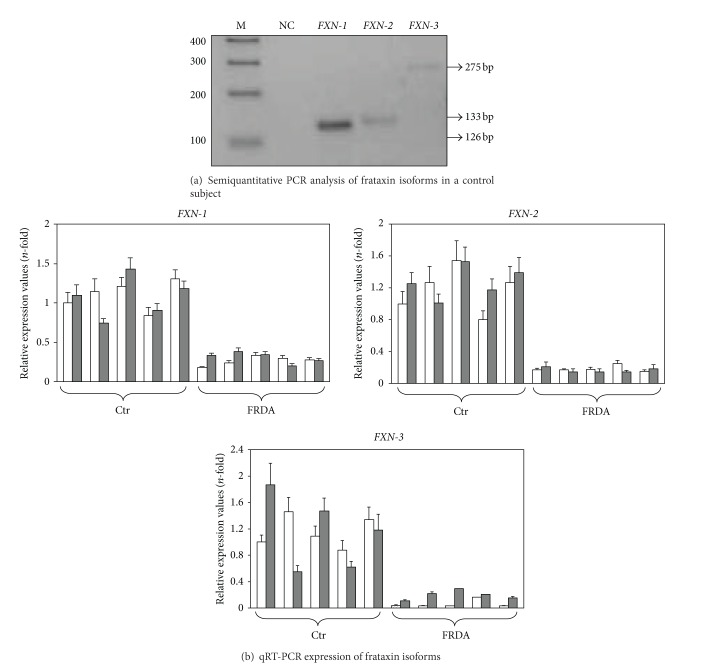
(a) Semiquantitative PCR analysis of frataxin isoforms in a control subject. Equal amount of a control subject cDNA was used for *FXN-1*, *FXN-2*, and *FXN-3* semiquantitative PCR analysis. PCR products were run in an agarose gel electrophoresis (2.5%). M: DNA ladder; NC: negative control. (b) qRT-PCR expression of frataxin isoforms. Frataxin isoform mRNA expression was analyzed by qRT-PCR in five healthy subjects (Ctr) and in five FRDA patients before (white columns) and after (grey columns) two-month tocotrienol supplementation. Data were normalized for two housekeeping genes, beta-actin and GAPDH; for each gene target, the normalized expression value of one control subject arbitrarily chosen was set to 1, and all other gene expression data were reported to that sample. Data are expressed as mean of technical triplicates ± SD and analyzed by ANOVA. Following tocotrienol supplementation, *FXN-3* mRNA increased 3.49-fold in FRDA patients (*p* ≤ 0.00000000342).

To compare the relative basal level of frataxin isoform gene expression, a semi-quantitative PCR was performed in a cDNA sample from a healthy control. The same amount of cDNA was amplified in separate wells by using the primer pairs specific for the three frataxin isoforms. The PCR products were then compared in a 2.5% agarose gel. As shown in Figure 2(a), *FXN-1* is way more expressed than *FXN-2* and *FXN-3*. These results were also confirmed by qRT-PCR, by comparing the number of cycles of the three isoforms after normalizing versus the housekeeping genes. Such analysis was carried out by means of the qbase^plus^ software. In particular, in healthy controls before treatment, we found that *FXN-2* and *FXN-3* mRNA expression was 4.72% and 2.47% of *FXN-1,* respectively. Our data are in agreement with those reported in the literature [4, 6]. 

### 3.3. Quantitative RT-PCR Gene Expression

Frataxin isoforms and *PPARG* gene expression were evaluated in FRDA patients and healthy control before and after tocotrienol treatment using qRT-PCR. As expected, all frataxin mRNA isoforms were less expressed in FRDA patients with respect to control subjects (Figure 2(b)), in line with data reported in literature [3]. In particular, in FRDA patients *FXN-1*, *FXN-2*, and *FXN-3* mRNAs were, respectively, 24.6%, 0.71%, and 0.11% compared to *FXN-1* of healthy subjects. 

FRDA patients treated with tocotrienol for two months showed 3.49-fold increase of *FXN-3* mRNA compared to FRDA patients before tocotrienol supplementation (*p* ≤ 0.00000000342) (Figure 2(b)), while *FXN-1* and *FXN-2* expression did not change after tocotrienol treatment (Figure 2(b)). Although tocotrienol treatment did not restore frataxin isoform mRNA expression to the levels of control subjects, in FRDA patients, we found an increase of 16% of total frataxin mRNA expression compared to FRDA patients before treatment. Tocotrienol treatment did not affect frataxin isoform gene expression in healthy controls.

**Figure 3:**
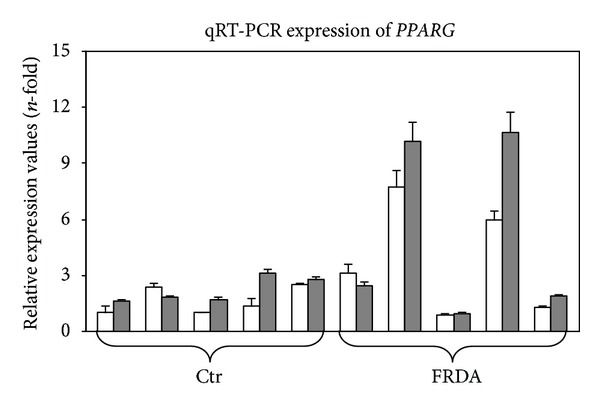
qRT-PCR expression of *PPARG*. Peroxisome proliferator-activated receptors-**γ* (PPARG)* mRNA expression was analyzed by qRT-PCR in five healthy subjects (Ctr) and in five FRDA patients before (white columns) and after (grey columns) two-month tocotrienol supplementation. Data were normalized for two housekeeping genes, beta-actin and GAPDH; for each gene target, the normalized expression value of one control subject arbitrarily chosen was set to 1, and all other gene expression data were reported to that sample. Data are expressed as mean of technical triplicates ± SD and analyzed by ANOVA.

Figure 3 shows the mRNA abundance of *PPARG* in FRDA patients and healthy controls before and after tocotrienol treatment. *PPARG* gene expression displayed a trend towards increase upon tocotrienol supplementation, although the increase was not statistically significant due to high variability within the groups.

### 3.4. Modelling of the Human FXN-2 and FXN-3 and of the Human NFS1/ISCU Complex

**Figure 4:**
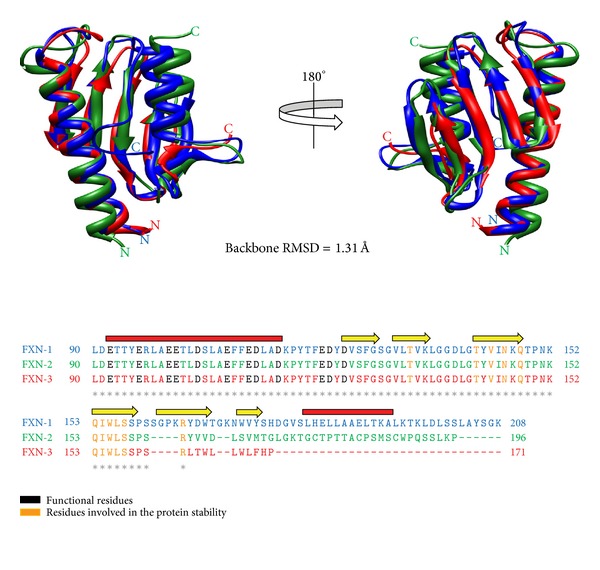
Structural comparison of human FXN-1 with FXN-2 and FXN-3. Sequence and structural alignment of FXN-1 (1EKG, depicted in blue) with the computed 3D models of FXN-2 and FXN-3 (colored in green and red, resp.). The secondary structure of FXN-1 is also reported along the structural alignment (helices: red line; beta sheets: yellow arrow). Functional residues of the anionic patch and residues involved in protein stability are highlighted in black and orange, respectively, and are conserved in all the isoforms.

The 3D model of the human FXN-2 and FXN-3 was built by adopting the structure of FXN-1 as a template.  Figure 4 shows the structural overlapping between isoform1, 2, and 3 and the corresponding structural alignment. Root mean square deviations (RMSD), as calculated on the protein backbone, are 1.31 Å and 1.32 Å among FXN-1 and FXN-2 and FXN-1 and FXN-3, respectively. FXN-1 and FXN-3 share identical residues with the exception of the C-terminus alpha helix. However, the two isoforms conserve residues that are correlated to the protein function: 12 charged residues involved in the anionic patch (E92, E96, E100, E101, E104, E108, E111, D112, D115, E121, D122, and D124) and 11 residues involved in the stability (T133, T142, V144, N146, Q148, Q153, I154, W155, L156, S157, and R165), which are well conserved in sequence, secondary, and tertiary structures. FXN-2 shares also 72 identical residues with FXN-1 in the functional region. In the C terminal portion, the two sequences differ in residue composition; however, residues in this part of the protein are enough to cover the C-terminus alpha helix of FXN-1. 

**Figure 5:**
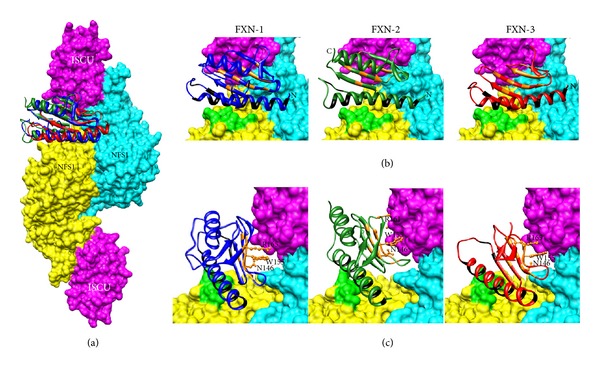
Docking of human FXN-1, FXN-2, and FXN-3 on the human tetrameric NFS1/ISCU complex. (a) The human iron-sulfur assembly complex was modeled adopting the *E. coli* counterpart as a template (PDB code: 3LVL). The NFS1/ISCU complex includes two copies of ISCU (surface representation in purple) and two copies of NFS1 (surface in cyan and yellow, resp.). The backbone of the three frataxin isoforms is depicted in blue (FXN-1), in green (FXN-2), and in red (FXN-3). The three backbones overlap with a pairwise root mean square deviation (RSMD) of about 1.3 Å. (b) Important residues essential for functional interaction are conserved in all isoforms and highlighted in black. Relevant interactions on the complex are highlighted in orange. Basic residues of NFS1 involved in the interaction with the anionic patch of frataxin (R218, R219, R221, and R223) are colored in light green. (c) Zooming on conserved residues among human frataxins was experimentally proven in yeast and humans to play a critical role in the interaction with the ISCU complex: W155 N146 and R165 (R161 in FXN-2 and FXN-3).

The 3D structure of the human complex NFS1/ISCU was computed on the basis of the available template of *E. coli* ISCS/ISCU (PDB code: 3LVL) [35]. Sequence identity between bacterial and human counterparts is 59% for the ISCS/NFS1 domains and 75% for the ISCU/ISCU domains. The values of sequence identity ensure that modelling results a reliable 3D structure (Figure 5). 

### 3.5. Docking of Human FXN-1, FXN-2, and FXN-3 to the Human NFS1/ISCU Complex

Mature human frataxin physically interacts with the Fe-S cluster biosynthesis machinery components NFS1 and ISCU [14]. Here, we investigated the binding capability of FXN-1, -2 and -3 to the complex in order to understand whether functional interaction is similar in the three isoforms. To this purpose, we modelled on the iron-sulfur assembly complex from *E.coli* the human counterparts and computed the 3D structure of the human tetrameric complex (see Figures 5(a) and 5(b)).

The anionic patch of frataxin is involved in the interaction with basic residues of NFS1 (R218, R219, R221, and R223, Figure 5(b)). The residues on the frataxin beta-sheets, in particular N146, W155, and R165, have been proven to be directly involved in the interaction with ISCU and also in protein stability (Figure 5(c)) [14, 37]. After docking of the FXN-3 model into the same complex, we found that all these relevant interactions are conserved (see zoomed portion of Figures 5(b) and 5(c)), thus corroborating the notion that the function of FXN-3 is preserved despite the lack of the C-terminus alpha helix. The docking of FXN-2 is also shown for the sake of comparison.

## 4. Discussion

Although frataxin role is not completely understood, frataxin deficiency has been suggested to sustain the increased oxidative stress, which is central in the pathogenesis of FRDA [38]. To date, there is still no effective cure for FRDA. As recently reported [39], one of the current therapeutic strategies in FRDA disease is the development of molecules aimed at increasing frataxin expression. Despite encouraging *in vitro* preliminary results, the effectiveness of these compounds as therapeutic agents requires more detailed studies to exclude unspecific and side effects in FRDA patients. Another therapeutic approach is aimed at counteracting oxidative stress. In this perspective, we focused our attention on tocotrienol, a potent antioxidant that is able to decrease a number of oxidative stress markers in FRDA patients (M. Marini, submitted paper). This encouraging result prompted us to investigate whether tocotrienol could modulate the expression of the different isoforms of frataxin mRNA. As previously reported, human mononuclear cells as well as fibroblasts, although they do not correspond to the cells affected in FRDA patients, represent a suitable in vitro model to study the expression of an ubiquitously expressed protein such as frataxin [5].

Here we report for the first time that tocotrienol induces, exclusively in FRDA patients, a significant 3.49-fold increase of the *FXN-3* isoform mRNA (Figure 2(b)). Such increase is specific, since it does not affect *FXN-1* or *FXN-2* (Figure 2(b)); therefore, the underlying mechanism(s) should include both an enhancement of gene transcription and/or of mRNA half-life and a regulation of differential splicing. No apparent relationship was found between the number of triplet repeats and frataxin expression and/or the amount of the tocotrienol-induced *FXN-3* increase. Although we are aware that the increase in *FXN-3* gene expression should be confirmed by an analysis of protein expression, at present, it is not possible to measure protein expression of the FXN-3 isoform because a specific antibody is not available. 

Marmolino and coworkers [40] showed that peroxisome proliferator-activated receptors-*γ* (PPARG) is involved in the upregulation of mRNA and protein frataxin expression in cell lines from FRDA patients. Tocotrienols both increase mRNA expression of PPAR receptors *α*, *γ*, and *δ* and, by directly binding their LBDs, induce an increase of their transcriptional activities [41]. *PPARG* gene expression was measured in both treated and untreated subjects in order to evaluate whether tocotrienol enhancement of *FXN-3* was mediated by an increase in *PPARG* expression. The high variability in *PPARG* expression among subjects did not allow a significant conclusion to be reached (Figure 3); however, as already pointed out, tocotrienol may activate PPARG transcriptional activities in a way that is independent of its increase [41].

On the other hand, tocotrienol may promote *FXN-3* mRNA expression through signalling pathways alternative to PPAR, as tocotrienols are able to modulate different targets at transcription, translation, and posttranslation levels [23].

Whether the tocotrienol-induced increase in *FXN-3* expression is biologically relevant obviously depends on the ability of the FXN-3 isoform to perform its tasks. To our knowledge, no functional and structural studies have been carried out for the FXN-3 isoform; therefore, we performed a structural analysis by means of bioinformatics tools. FXN-3 and FXN-1 share a 93% protein sequence identity; from the comparison of their sequences, the residues known to be correlated with protein function [8] and with stability [37] were found to be well conserved in sequence, secondary, and tertiary structures (Figure 4). 

By modeling of the iron-sulphur assembly complex from *E. coli* and computing from it the 3D structure of the human tetrameric complex, we found that all relevant interactions are conserved between FXN-1, FXN-2, and FXN-3 (see zoomed portion of Figure 5), with comparable docking binding energy. Despite the partial or total lack of the C-terminus alpha helix, these data show that both FXN-2 and FXN-3 not only have an identity of structure compared to FXN-1 but also have the same ability to interact with NFS1/ISCU. These bioinformatics data show for the first time that both FXN-2 and FXN-3 may have a biological function and can be considered a starting point for future studies aimed at confirming the function of both FXN-2 and FXN-3 isoforms by using the purified protein.

## 5. Conclusions

Our results demonstrate, for the first time, (i) that tocotrienol induces in FRDA patients the upregulation of the *FXN-3* mRNA isoform and (ii) that FXN-3 and FXN-2 proteins share the same 3D structure as FXN-1 and are apparently able to complex with NFS1/ISCU, thus suggesting that they may play the same functions. Since basal level of *FXN-3* is very low, its 3.49-fold increase in tocotrienol-treated patients was not enough to reach frataxin mRNA amounts enabling normal cellular functions [3]. 

The mechanism by which tocotrienol enhances *FXN-3* mRNA levels should be further investigated. In fact, it has been reported that SRF and TFAP2 iron-dependent transcription factors regulate frataxin expression [42] and that tocotrienol stimulates PPARG activity dose dependently [41]. Therefore, we are planning to evaluate in FRDA cell lines whether higher tocotrienol concentrations could further enhance frataxin mRNA levels, in PPARG-dependent or independent manner. Cell culture studies will also allow the evaluation of variations in frataxin protein levels, which is almost impossible to perform in mononuclear cells, which are poor frataxin producers. This will hopefully enable the use of tocotrienol not only as antioxidant therapy but also as inducer of frataxin expression.

## Supplementary Material

Table 1: Reports the primer sequence (left and right), amplicon length (bp) and the Unigene Accession number of all genes studied in qRT-PCR. Beta-Actin and GADPH genes were used for normalization purposes.

## Abbreviations

FRDA: Friedreich's ataxia
FXN-1: Frataxin isoform 1
FXN-2: Frataxin isoform 2
FXN-3: Frataxin isoform 3
NFS1: Iron-sulfur cluster assembly protein
ISCU: Iron-sulfur cluster assembly scaffold protein
PPARG: Peroxisome proliferator-activated receptors-*γ*.
