# Supplementary material for: Frataxin mRNA Isoforms in FRDA Patients and Normal Subjects: Effect of Tocotrienol Supplementation
Source: Biomed Res Int. 2013 Sep 23;2013:276808. doi: 10.1155/2013/276808 (PMC3794619; doi:10.1155/2013/276808)
Supplement: Supplementary file 1 — Table 1: Reports the primer sequence (left and right), amplicon length (bp) and the Unigene Accession number of all genes studied in qRT-PCR. Beta-Actin and GADPH genes were used for normalization purposes. [file 276808.f1.pdf]

Table 1: Primer sequence and amplicon length of the genes studied with real-time PCR. Beta-actin and GADPH genes were used for normalization purposes.

**Unigene accession no.**	**Gene**	**Left Primer**	**Right Primer**	**Amplicon length (bp)**
Hs.520640	*Beta actin*	TGTGGCATCCACGAAACTAC	TGATCTTGATCTTCATTGTGCT	175
Hs.544577	*GADPH*	GGCCTCCAAGGAGTAAGACC	CTGTGAGGAGGGGAGATTCA	130
Hs.20685	*FXN-1*	GATGTCTCCTTTGGGAGTGG	ACGCTTAGGTCCACTGGATG	126
Hs.20685	*FXN-2*	GATGTCTCCTTTGGGAGTGG	CGCTTAGGTCCACTACATACCTG	133
Hs.20685	*FXN-3*	CCTTGCAGACAAGCCATACA	CTTCGTTGCTCACTTGCTGA	275
Hs.162646	*PPARG*	CATAAAGTCCTTCCCGCTGA	ACCTCTTTGCTCTCCTCCTG	165
